# Fabrication of Al_2_O_3_/ZnO and Al_2_O_3_/Cu Reinforced Silicone Rubber Composite Pads for Thermal Interface Materials

**DOI:** 10.3390/polym13193259

**Published:** 2021-09-24

**Authors:** Seokkyu Jang, Eun Ji Choi, Han Jin Cheon, Won Il Choi, Woon Seo Shin, Jong-Min Lim

**Affiliations:** 1 Department of Chemical Engineering, Soonchunhyang University, 22 Soonchunhyang-ro, Shinchang-myeon, Asan-si 31538, Chungcheongnam-do, Korea; wkdtjrrb0803@naver.com; 2 Doosung Industrial Co., Ltd., 36 Ansantekom 1-gil, Sangnok-gu, Ansan-si 15523, Gyeonggi-do, Korea; cheon7609@dsic21.com; 3 Department of Electronic Materials and Devices Engineering, Soonchunhyang University, 22 Soonchunhyang-ro, Shinchang-myeon, Asan-si 31538, Chungcheongnam-do, Korea; chldmswl6502@naver.com; 4 Center for Convergence Bioceramic Materials, Korea Institute of Ceramic Engineering and Technology, 202, Osongsaengmyeong 1-ro, Osong-eup, Heungdeok-gu, Cheongju 28160, Chungbuk, Korea

**Keywords:** thermal pads, thermal interface material, composite, heat transfer, aluminum oxide, zinc oxide, copper

## Abstract

Thermal interface materials (also known as thermal pads) are widely used as a crucial part to dissipate heat generated in miniaturized and integrated electronic components. Here, we systematically investigated the effects of small ceramic and metallic powders in rubbery thermal composite pads with a high content of aluminum oxide filler on the thermal conductivity of the composite pads. We optimized the compositions of aluminum oxide fillers with two different sizes in a polydimethylsiloxane (PDMS) matrix for rubbery composite pads with a high thermal conductivity. Based on the optimized compositions, zinc oxide powder or copper powder with an average size of 1 μm was used to replace 5 μm-sized aluminum oxide filler to examine the effects of the small ceramic and metallic powders, respectively, on the thermal conductivity of the composite pads. When zinc oxide powder was used as the replacement, the thermal conductivity of the rubbery composite pads decreased because more air bubbles were generated during the processing of the mixed paste with increased viscosity. On the other hand, when the copper powder was used as a replacement, a thermal conductivity of up to 2.466 W/m·K was achieved for the rubbery composite pads by optimizing the mixing composition. SEM images and EDS mapping confirmed that all fillers were evenly distributed in the rubbery composite pads.

## 1. Introduction

Thermal management has become one of the most important issues in preventing the thermal failure of electronic devices because more heat is generated as electronic devices become miniaturized and integrated. Personalized electronic devices, such as smartphones and wearable devices, have various functionalities, resulting in integrated and miniaturized electronic components with a larger power consumption. The demand for thinner, lighter, smaller, and waterproof personalized electronic devices is increasing. According to a US statistic, 55% of malfunctions and performance degradation of electronic devices are due to heat generation, which has emerged as an important issue in terms of the heat resistance and lifespan extension of electronic devices [1,2,3]. Since most of the personalized electronic devices do not use a cooling fan, thermal interface materials (also known as thermal pads) are widely used to enable low-thermal-resistance contact of heated electronic components with a metal-plate heat sink. A soft and deformable thermal pad is sandwiched between two stiff planes to fill voids.

To prepare soft thermal pads with high thermal conductivity, composites made of thermally conductive fillers in a polymer matrix have been widely studied [1,2,3]. Although the polymer itself is thermally non-conductive (0.17‒0.35 W/m∙K), adding conductive fillers can improve both heat conduction performance and mechanical properties of the composite, while maintaining the advantages of good formability, low manufacturing cost, and a good appearance of the final product. Various conductive fillers including metal, ceramic, carbon, and other inorganic particles have been studied to prepare thermal pads [4,5,6,7,8,9,10,11,12,13,14,15]. Although thermal pads with metal or carbon fillers exhibit high thermal conductivity, electric conductivity is also high when the concentration of metal or carbon fillers exceeds the threshold value [16,17,18,19,20,21]. On the other hand, thermal pads with ceramic fillers have a relatively high thermal conductivity while maintaining low electric conductivity even at high filler concentrations. Thus, ceramic fillers are widely used to dissipate heat in electronic devices [12,13,14,15,22,23]. Among these ceramic fillers, aluminum oxide is the most widely used in commercial thermal pads as a conductive filler due to its low cost, high chemical stability, relatively high thermal conductivity (about 30 W/m·K), and low electric conductivity [14,15,23]. Heat transfer in ceramic fillers occurs due to the vibration of the phonon lattice. The heat conduction can be increased by factors such as a low element mass, strong attraction between atoms, high coordination between lattices, and high crystallinity [24,25]. Although various studies have prepared thermal pads with high conductivity, the effects of heterogeneous fillers on the thermal conductivity of thermal pads with high contents of aluminum oxide still need to be systematically explored.

Here we systematically evaluated the effects of small ceramic or metallic powder additions on the thermal conductivity of rubbery composite pads with a high content of aluminum oxide. Polydimethylsiloxane (PDMS) has been widely studied to prepare functional composites by embedding various fillers as it guarantees a conformal contact with uneven surfaces due to its flexibility and elasticity [26]. Thus, it was used in this study as the polymer matrix. Conformal contact with a heat source can enhance the heat conduction effect by avoiding air void formation at the interface. Since aluminum oxide is widely adopted in the industry to prepare thermal pads, we first systematically optimized the composition of aluminum oxide powder in the silicon rubber composite pads. Aluminum oxide powder with two different sizes (i.e., 70 μm and 5 μm) was used to prepare the thermal pads, because thermal conductivity could be enhanced by using micro-sized powders to make a composite with a powder mixture processing method [27,28]. Small ceramic or metal powders were then added to replace the 5 μm-sized aluminum oxide powder to examine the effect of the replacement on the thermal conductivity of the thermal pads and optimize the composition. Irregular zinc oxide powder (60 W/m·K) and spherical copper powder (400 W/m·K) were used as small ceramic and metal fillers, respectively, because of their higher thermal conductivity than aluminum oxide powder (30 W/m·K). This systematical study of thermal conductivity of rubbery composite pads with heterogeneous fillers will expedite scientific understandings and industrial applications of thermal interface materials, which can contribute to enhancing the performance and speed of electronic devices and batteries by preventing thermal malfunction and thermal throttling [29,30,31].

## 2. Materials and Methods

### 2.1. Materials

Polydimethylsiloxane (PDMS, SYLGARD^TM^ 184 Kit) was purchased from Dow-corning (Midland, MI, USA). Spherical aluminum oxide powder (70 μm and 5 μm, 99.9%), irregular zinc oxide powder (1 μm, 99.9%), and spherical copper powder (1 μm, 99.9%) were purchased from Avention (Incheon, Korea). The physical properties (i.e., size, density, and thermal conductivity) of the powders are listed in Table 1. Fluorine release film (P 50) was obtained from FNSTech (Ansan, Korea). All materials were used without further purification.

### 2.2. Fabrication of Rubbery Composite Pads

**Scheme 1:**
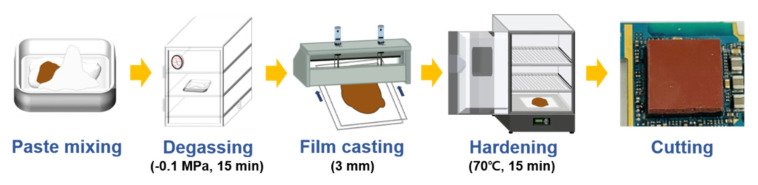
Schematic illustration of the fabrication procedure and a digital camera image of the rubbery composite pad.

As illustrated in Scheme 1, the fabrication procedure of rubbery composite pads consisted of paste mixing, degassing, film casting, curing, and cutting. The PDMS and powders used as matrix and filler, respectively, were mixed according to the designed compositions. The mixed paste was degassed using a vacuum desiccator (JEIO TECH, Daejeon, Korea) at −0.1 MPa for 15 min. Degassed mixed paste was poured onto a fluorine release film, which was placed on a hard, flat board. Another fluorine release film was slowly covered over the mixed paste to minimize bubble formation. The thickness of the mixed paste was adjusted to 3 mm using a micro film applicator (MTI corporation, Richmond, CA, USA). The mixed paste film was then cured at 70 °C for 15 min using a convection oven (Jongro industrial corporation, JRFD-803, Seoul, Korea). The rubbery composite pad was then cut into the desired shape.

### 2.3. Characterization

After the degassed mixed paste was poured onto a fluorine release film, the viscosity of the residual degassed mixed paste without curing was measured using a viscometer (Brookfield, DV Next HB series, Middleborough, MA, USA) with a cone-and-plate-type spindle (CPA-52Z) at 1 rpm and 25 °C. Thermal conductivity was measured at room temperature using a TCi thermal conductivity analyzer (C-THERM, Fredericton, NB, Canada). The rubbery composite pads needed to be at least 40 mm × 40 mm × 3 mm for the thermal conductivity measurements. A 500 g weight was placed on the sample, which was then placed on the sensor, to ensure conformal contact between the sensor and the sample. Thermal conductivity measurements were repeated 9 times for each sample. The instrument for thermal conductivity measurements was displayed to the third decimal place. After curing, the rubbery composite pads were sliced using a razor blade and coated with a thin layer of Pt. These rubbery composite pads were imaged using a high-resolution scanning electron microscope (HR FE-SEM, Tescan, MIRA3-LMH, Brno, Czech Republic) equipped with an energy dispersive X-ray analyzer (EDS). Elemental mappings of Si, Al, Zn, and Cu were performed using the EDS. The electrical insulating performance of the rubbery composite pads was tested using a surface resistance checker (Surpa, model 385, Shenzhen, China).

## 3. Results and Discussion

### 3.1. Preparation of Al_2_O_3_ Reinforced Rubbery Composite Pads

The composition of the aluminum oxide powder in the silicon rubber was optimized to prepare the composite pads for the thermal interface materials. As shown in Table 2, we used aluminum oxide powder with two different sizes (i.e., 70 μm and 5 μm) as the filler, and PDMS as the polymeric matrix. Spherical aluminum oxide powders are most widely adopted in the industry to prepare thermal pads due to their low cost, high chemical stability, relatively high thermal conductivity (30 W/m·K), and low electric conductivity [14,23]. There is a gap between particles when particles with similar sizes are used as fillers, which limits the ratio of filler in a composite. To fill the space between larger particles with smaller particles, aluminum powders with two different sizes can be used to prepare composite pads [28,34]. Since the surface area of a spherical particle is smaller than that of a non-spherical particle, the viscosity of a mixed paste is lowered when spherical particles are used. When the viscosity of the mixed paste is low, the formation of bubbles during the paste mixing process can be reduced. Since the thermal conductivity of air is extremely low (0.026 W/m·K), overall conductivity is reduced when air bubbles are formed in a composite pad. PDMS is widely adopted as a polymer matrix to prepare rubbery composite pads because of its good physical properties including flexibility, elasticity, optical transparency, and biocompatibility [26,36]. Although the thermal conductivity of PDMS is relatively low (0.27 W/m·K), composites based on PDMS can be used as soft thermal pads. The space between an electronic component and a heat sink is filled with a soft thermal pad, which enhances the heat transfer by prevent air void formation. The flexibility and elasticity of PDMS enables a conformal contact of a rubbery composite pad with uneven surfaces, which can minimize the formation of air void at the interface.

The ratio of the curing agent in the paste mixture is reduced to prepare softer composite pads. When powders are added to a mixed paste, the viscosity of the paste and the stiffness of the cured composite pad are increased. In both cases, the overall thermal conductivity of the composite pad is low because the chance to form air bubbles in the composite pad is increased. When we used the base elastomer and curing agent at a ratio of 10:1, partial curing occurred during the mixing and the degassing process, even at room temperature. In addition, the cured composite pad was stiff and brittle. Although it is usually recommended to use a base elastomer and a curing agent at a ratio of 10:1 when the PDMS mixture is prepared, partial curing at room temperature can be retarded, and the stiffness of the cured PDMS can be lowered by reducing the amount of curing agent in the mixture. To prepare a softer composite pad and to prevent partial curing during the process at room temperature, the ratio of the base elastomer and curing agent was fixed to 19:1 throughout the experiment.

The ratio of larger and smaller aluminum oxide powders was changed systematically, while the content of the PDMS matrix was fixed to 20 or 15 wt%, as shown in Table 2. Since the thermal conductivity of the filler is higher than that of the PDMS matrix, the ratio of the PDMS matrix should be decreased to achieve a high thermal conductivity. In addition, the filler should be mixed well enough to achieve a uniform heat distribution and physical properties throughout the thermal pad. When the content of the PDMS matrix was lower than 15 wt%, in some cases the fillers were not mixed well enough to prepare composite pads. The ratio of 70 μm-sized aluminum oxide to 5 μm-sized aluminum oxide was changed systematically, while the content of the PDMS matrix was fixed to 20 wt% in type A–C and 15 wt% in type D–F, as shown in Table 2. It was noteworthy that the amount of the larger aluminum oxide powder was the same or higher than that of the smaller aluminum oxide powder, because smaller fillers were used to fill the space between larger fillers. 

Bubble formation should be minimized throughout the composite preparation processes because some can create pores in the composite pad, even after curing. To measure the thermal conductivity accurately, pores in the composite pads should be minimized. After mixing the paste with the designed ratio, as shown in Table 2, the mixed paste was degassed using a vacuum desiccator. The pressure should be dropped gradually to prevent the splash of the mixed paste on the wall of the desiccator, which can result in the generation of air bubbles. After the vacuum gauge reached −0.1 MPa, the mixed paste was degassed for 15 min. When the degassing time was shorter, lots of bubbles were not removed by the degassing process, with some of them creating pores in the composite pad, even after curing. When the degassing time was too long, the mixed paste was partially cured, even at room temperature. In our experiments, the degassing time was optimized to 15 min.

The modified transient plane source method was used to measure thermal conductivity with the TCi thermal conductivity analyzer. In this method, thermal effusivity was measured and thermal conductivity was then calculated using the following Equation:

(1) $$e=\sqrt{{k\rho c}_{p}}$$

where e was the thermal effusivity (W∙$S^{\frac{1}{2}}$/$m^{2}\cdot K$), k was the thermal conductivity (W/m∙K), ρ was the density (kg/m^3^), and $c_{p}$ was the specific heat capacity (J/kg∙K). The thickness of the composite pad was adjusted to 3 mm using a micro film applicator, because the thickness should be at least 2 mm for thermal conductivity measurement with the TCi thermal conductivity analyzer. Stabilization was performed for 60 s before each measurement.

**Figure 1:**
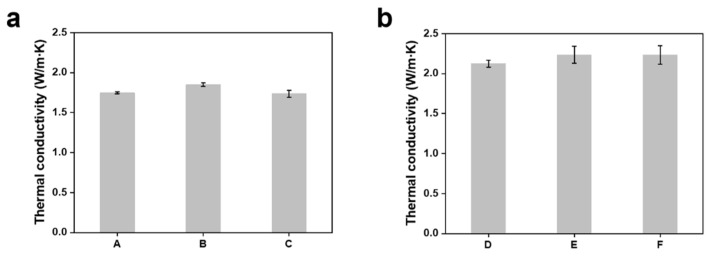
Thermal conductivities of thermal pads using aluminum oxide powder with (**a**) 20 wt% PDMS or (**b**) 15 wt% PDMS. The bar graph illustrating the mean and standard deviation (error bars).

The thermal conductivity of the aluminum oxide reinforced rubbery composite pad is summarized in Figure 1. The designed composition of the PDMS matrix and aluminum oxide fillers in each type of sample is shown in Table 2. When the content of the PDMS matrix was 20 wt%, type B had the highest thermal conductivity of 1.849 W/m·K, as shown in Figure 1a. When the content of the PDMS matrix was 15 wt%, type E and type F had higher thermal conductivities than type D. The average thermal conductivities of type E and type F were 2.232 W/m·K and 2.236 W/m·K, respectively. When the error bar was considered, the thermal conductivities of type E and F had almost the same value. The ratio of aluminum oxide powders in type E was similar to that in type B, which had the highest thermal conductivity when the content of the PDMS matrix was 20%. As a result, the compositions of type B and type E were used for additional experiments, due to their high thermal conductivities when the content of the PDMS matrix was 20 wt% or 15 wt%. 

**Figure 2:**
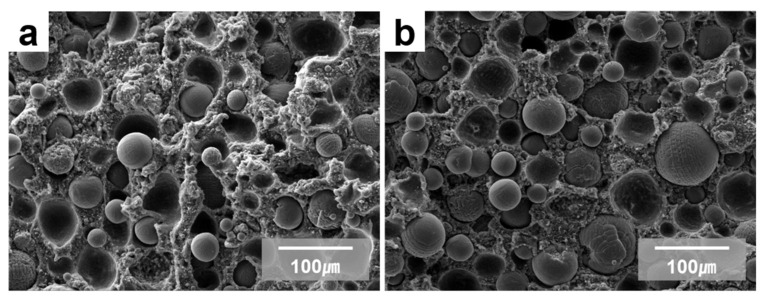
Cross-sectional SEM images of thermal pads using aluminum oxide powder of (**a**) type B and (**b**) type E.

**Figure 3:**
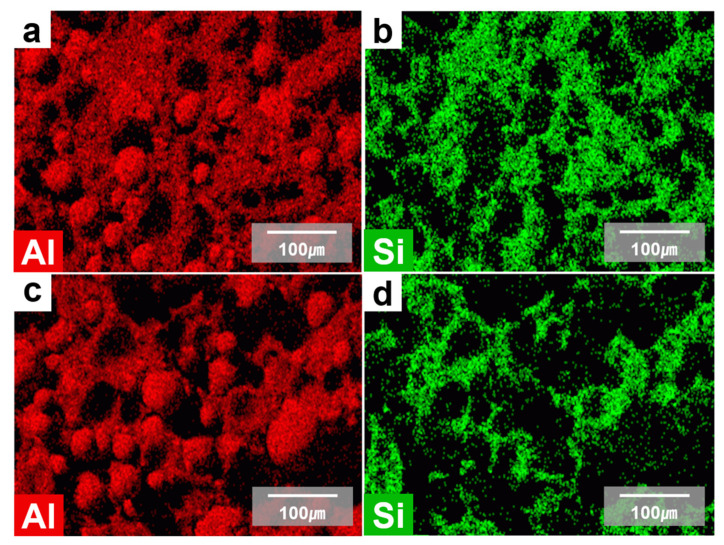
EDS mapping of thermal pads using aluminum oxide powder of (**a**,**b**) type B and (**c**,**d**) type E. Al and Si are represented by red and green colors, respectively.

Aluminum oxide fillers and the PDMS matrix were evenly distributed in the rubbery composite pad, as confirmed by cross-sectional SEM images (Figure 2) and EDS mapping (Figure 3). As shown in the cross-sectional SEM images of Figure 2, aluminum oxide fillers were evenly distributed in the rubbery composite pads without aggregation of fillers. Most voids in the cross-sectional SEM image originated from the detachment of the aluminum oxide powder during razor blade cutting for cross-sectional SEM sample preparation. The detailed distribution of the aluminum oxide filler and PDMS matrix were investigated using EDS mapping of Al and Si elements, respectively (Figure 3). The results confirmed that both the aluminum oxide filler and PDMS matrix were evenly distributed in the rubbery composite pads. The black regions were voids, which mainly originated from the detachment of aluminum oxide fillers during razor blade cutting for sample preparation.

### 3.2. Preparation of Al_2_O_3_/ZnO Reinforced Rubbery Composite Pads

To examine the effect of a small ceramic powder addition on thermal conductivity, a portion of 5 μm-sized aluminum oxide powder was replaced with irregular shaped zinc oxide powder with an average size of 1 μm. As discussed earlier, compositions of type B and type E in Table 2 exhibited high thermal conductivity when contents of the PDMS matrix were 20 wt% and 15 wt%, respectively. As a result, a portion of the 5 μm-sized aluminum oxide powder in type B and type E was replaced with same amount of 1 μm-sized zinc oxide powder, while the weight ratio of the PDMS matrix to the 70 μm-sized aluminum oxide powder was fixed (Table 3). Previously, Mu et al. [34] have reported that thermal conductivity could be enhanced when zinc oxide powders with different sizes are used as fillers in silicone rubber matrix. When a smaller zinc oxide powder was used as a filler, the thermal conductivity of the composites could be enhanced because smaller fillers could exist between larger-sized fillers to make more heat transfer paths [34]. In addition, the thermal conductivity of zinc oxide powder (60 W/m·K) is higher than that of aluminum oxide (30 W/m·K). Therefore, we hypothesized that the thermal conductivity might be increased if we replaced the 5 μm-sized aluminum oxide powder with 1 μm-sized zinc oxide powder.

**Figure 4:**
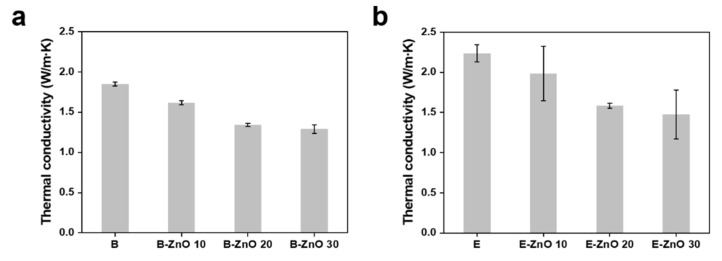
Thermal conductivities of thermal pads using aluminum oxide and zinc oxide powder with (**a**) 20 wt% PDMS or (**b**) 15 wt% PDMS. The bar graph illustrates the mean and standard deviation (error bars).

However, the thermal conductivity of the composite pad decreased when the content of the zinc oxide powder increased, as shown in Figure 4. As shown in Figure 4a, type B-ZnO 10 had a thermal conductivity of 1.618 W/m·K, which was the highest value besides type B without zinc oxide powder. Similarly, type E-ZnO 10 had a thermal conductivity of 1.984 W/m·K, which was the highest value besides type E without zinc oxide powder, as shown in Figure 4b. Thermal conductivity reduced as more ZnO powder was used for replacement, which was contrary to our hypothesis.

The thermal conductivity reduction originated from an increase in air bubble formation due to the increased viscosity of the mixed paste. We noticed that it took a longer time to mix because of the thicker paste when the irregular-shaped zinc oxide powder was used for replacement. The viscosities of the uncured mixed paste of type B and type B-ZnO 30 were 370,700 cP and 630,200 cP, respectively. After 5 μm-sized aluminum oxide was replaced with 1 μm-sized zinc oxide in type B, the viscosity of the uncured paste increased 1.7 times. Similarly, the viscosities of the uncured mixed paste of type E and type E-ZnO 30 was 1,720,000 cP and 3,258,000 cP, respectively. After the 5 μm-sized aluminum oxide was replaced with 1 μm-sized zinc oxide in type E, the viscosity of the uncured paste increased 1.9 times. A higher viscosity of paste induced more air bubble generation during the mixing process. In addition, the entrapped air bubbles suffered more resistance to removal during the degassing process. Since the thermal conductivity of air was extremely low (0.026 W/m·K), the increase in air bubble formation resulted in a reduction of the overall thermal conductivity of the rubbery composite pad.

**Figure 5:**
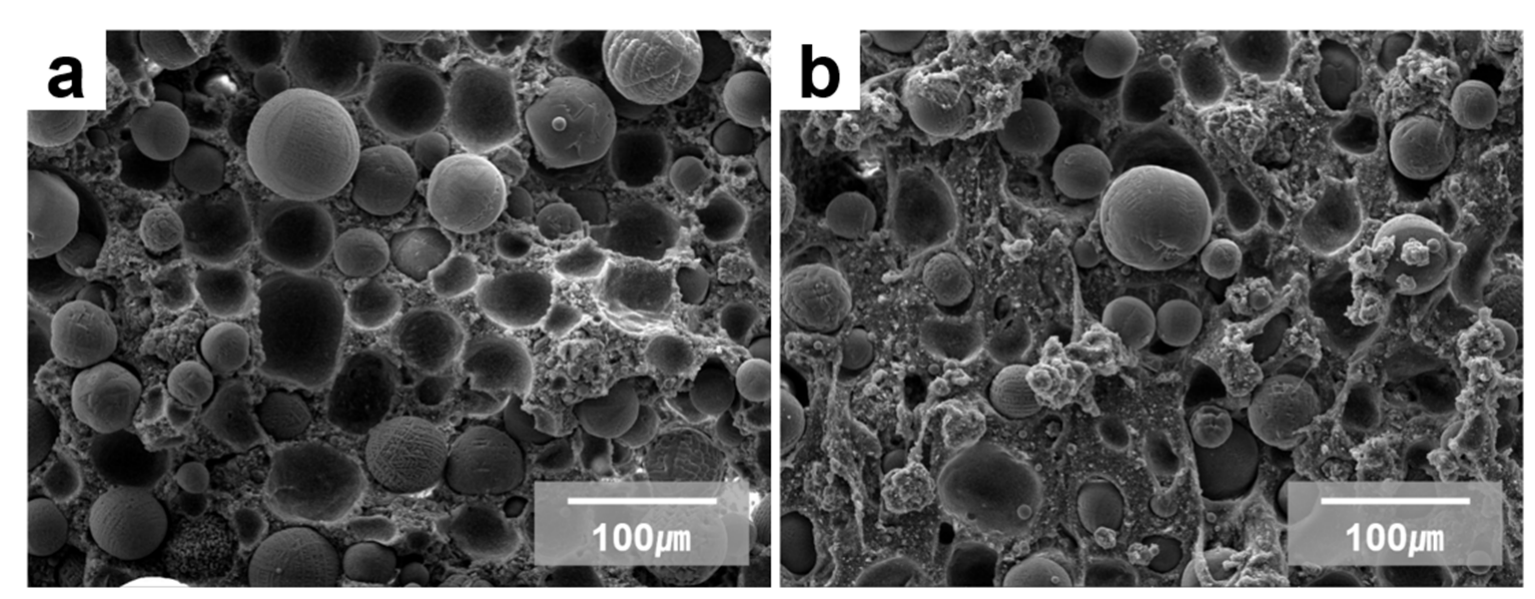
Cross-sectional SEM image of thermal pads using aluminum oxide and zinc oxide powder of (**a**) type B-ZnO 10 and (**b**) type E-ZnO 10.

**Figure 6:**
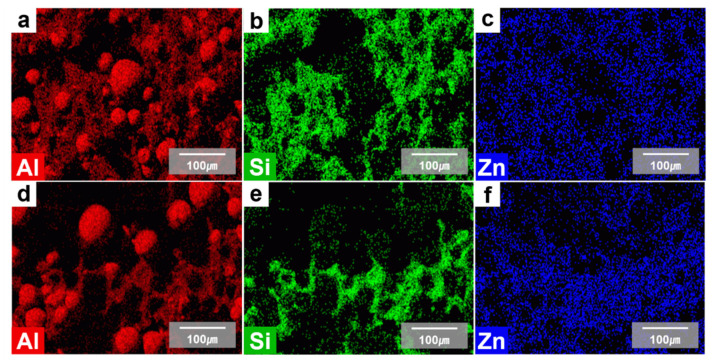
EDS mapping of thermal pads using aluminum oxide and zinc oxide powder of (**a**–**c**) type B-ZnO 10 and (**d**–**f**) type E-ZnO 10. Al, Si, and Zn are represented by red, green, and blue colors, respectively.

Aluminum oxide and zinc oxide fillers were evenly distributed in the PDMS matrix, as confirmed by cross-sectional SEM images (Figure 5) and EDS mapping (Figure 6). As shown in the cross-sectional SEM images of Figure 5, fillers were evenly distributed in the rubbery composite pads without aggregation. Most voids in the cross-sectional SEM images originated from the detachment of fillers during razor-blade cutting for cross-sectional SEM sample preparation. Detailed distributions of aluminum oxide, zinc oxide, and the PDMS matrix were examined using EDS mapping of Al, Zn, and Si elements, respectively (Figure 6). Results confirmed that the aluminum oxide filler, zinc oxide filler, and the PDMS matrix were evenly distributed in the rubbery composite pads.

### 3.3. Preparation of Al_2_O_3_/Cu Reinforced Rubbery Composite Pads

To investigate the effect of a small metal powder addition on thermal conductivity, a portion of 5 μm-sized aluminum oxide powder was replaced with spherical-shaped copper powder with an average size of 1 μm. Similar to the previous zinc oxide case, a portion of 5 μm-sized aluminum oxide powder in type B and type E was replaced with the same amount of 1 μm-sized copper powder, while the weight ratio of the PDMS matrix to 70 μm-sized aluminum oxide powder was fixed (Table 4). Since the thermal conductivity of copper (400 W/m·K) is much higher than that of aluminum oxide (30 W/m·K), we hypothesized that the thermal conductivity might be increased if the 5 μm-sized aluminum oxide powder was replaced with 1 μm-sized copper powder.

**Figure 7:**
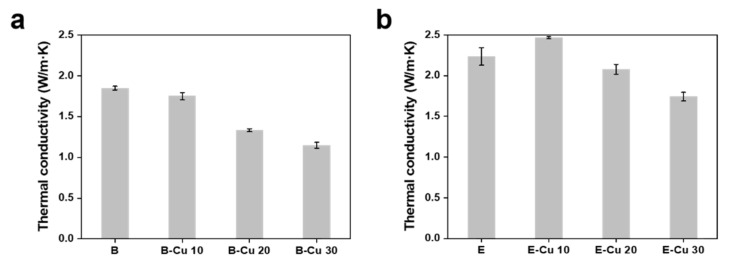
Thermal conductivity of thermal pads using aluminum oxide and copper powder with (**a**) 20 wt% PDMS or (**b**) 15 wt% PDMS. The bar graph illustrating the mean and standard deviation (error bars).

We could find the optimal composition to enhance the thermal conductivity of the rubbery composite pad made of aluminum oxide filler, copper filler, and PDMS matrix. Although spherical copper powder had a much higher thermal conductivity (400 W/m·K) than aluminum oxide powder (30 W/m·K), the thermal conductivity of the rubbery pad with 20 wt% PDMS matrix was reduced when aluminum oxide powder was replaced with copper powder (Figure 7a). As shown in Figure 7a, type B-Cu 10 had a thermal conductivity of 1.750 W/m·K, which was the highest value besides type B without Cu powder. As shown in Figure 7b, on the other hand, the thermal conductivity of the rubbery composite pads with alumina and copper fillers could only be enhanced by type E-Cu 10, where 10% of aluminum oxide filler was replaced with copper filler with 15 wt% PDMS matrix. The thermal conductivity of type E-Cu 10 was 2.466 W/m·K, which was the highest thermal conductivity in this experiment. Although the thermal conductivity of the copper powder was higher than those of the other powders in this study, the density of the copper powder was also higher, as summarized in Table 1. To achieve a high thermal conductivity, the rubbery composite must have enough heat transfer paths. Because the density of copper is higher than that of aluminum oxide, the volume fraction occupied by the filler in the composite pad was reduced when the aluminum oxide filler was replaced with the same weight fraction of copper. As a result, the rubbery composite pad with aluminum oxide and copper fillers had the optimal composition to enhance the overall thermal conductivity. 

**Figure 8:**
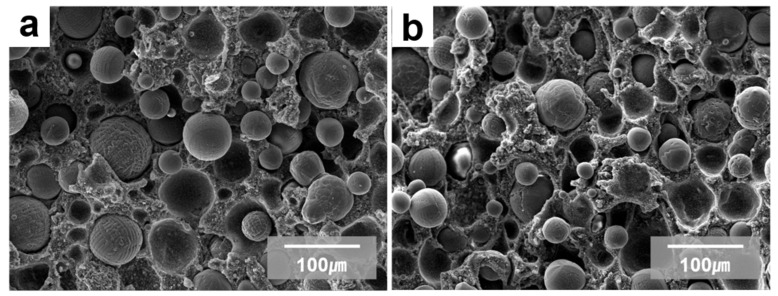
Cross-sectional SEM images of thermal pads using aluminum oxide and copper powder of (**a**) type B-Cu 10 and (**b**) type E-Cu 10.

**Figure 9:**
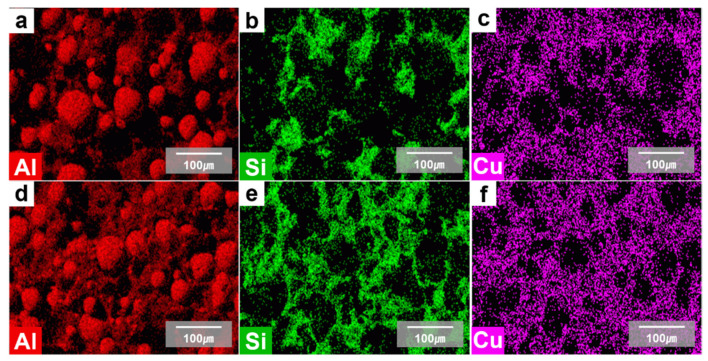
EDS mapping of thermal pads using aluminum oxide and copper powder of (**a**–**c**) type B-Cu 10 and (**d**–**f**) type E-Cu 10. Al, Si, and Cu are represented with red, green, and purple colors, respectively.

Aluminum oxide and copper fillers were evenly distributed in the PDMS matrix, as confirmed by cross-sectional SEM images (Figure 8) and EDS mapping (Figure 9). As shown in the cross-sectional SEM images of Figure 8, fillers were evenly distributed in the rubbery composite pads without aggregation. Most voids in the cross-sectional SEM images originated from the detachment of the fillers during razor blade cutting for cross-sectional SEM sample preparation. Detailed distribution of aluminum oxide, copper, and PDMS matrix could be confirmed by EDS mappings of Al, Cu, and Si elements, respectively (Figure 9). Results confirmed that the aluminum oxide filler, copper filler, and PDMS matrix were evenly distributed in the rubbery composite pads.

Most of the thermal interface materials used in electronic devices should possess good insulating properties and elasticity. The electric insulating performances of type E, E-ZnO 10, and E-Cu 10 were tested using a surface resistance checker, because they exhibited high thermal conductivity. The test results were “insulative”, which means that the rubbery composite pads possess good insulating properties. In addition, the rubbery composite pads could be easily bent by fingers, which means they are elastic enough to be utilized for thermal interface materials.

## 4. Conclusions

We systematically evaluated the effects of small ceramic and metallic powders on the thermal conductivity of rubbery thermal pads with high contents of aluminum oxide filler. First, the mixing ratio of the spherical aluminum oxide filler (30 W/m·K) at two different sizes (i.e., 70 μm and 5 μm) to PDMS matrix (0.27 W/m·K) was controlled systematically in order to optimize the thermal conductivity of the rubbery composite pads with high contents of aluminum oxide. Based on the optimal composition of rubbery composite pads made of aluminum oxide fillers and PDMS matrix, 5 μm-sized aluminum oxide powder was replaced with irregular zinc oxide (60 W/m·K) or spherical copper (400 W/m·K) powder with an average size of 1 μm. All fillers were evenly distributed in the PDMS matrix without any aggregations, as confirmed by cross-sectional SEM images and EDS mapping. In the case of zinc oxide powder being used, the thermal conductivity reduced as the ratio of zinc oxide was increased, even though the thermal conductivity of zinc oxide was higher than that of aluminum oxide. Since the viscosity of the uncured paste was increased by replacing spherical aluminum oxide powder with irregular zinc oxide powder, more air bubbles were generated in the rubbery composite pads. Because air bubbles have an extremely low thermal conductivity (0.026 W/m·K), the overall thermal conductivity of the rubbery composite pads decreased. In the case of copper powder being used, the maximal thermal conductivity value that could be achieved was 2.466 W/m·K when 10 wt% of aluminum oxide powder was replaced with copper powder, and 15 wt% PDMS was used (type E-Cu 10). Results of this systematical study on the thermal conductivity of rubbery composite pads with heterogeneous fillers can be utilized to enhance the performance of thermal interface materials widely used in various fields including electronics, battery, aerospace, military, and next-generation automobiles. 

## Figures and Tables

**Table 1:** Properties of PDMS [32], aluminum oxide [23,33], zinc oxide [23,34], and copper [35] powder.

	PDMS	Al_2_O_3_ Powder		ZnO Powder	Cu Powder
Size (μm)	-	70	5	1	1
Density (g/cm^3^)	0.982	3.87	3.74	5.47	8.9
Thermal conductivity (W/m∙K)	0.27	30		60	400

**Table 2:** Mixing ratio using aluminum oxide powder with different sizes.

Type	Matrix (PDMS)				Filler			
	Base		Curing Agent		70 μm Al_2_O_3_		5 μm Al_2_O_3_	
	wt%	vol%	wt%	vol%	wt%	vol%	wt%	vol%
A	19	46.73	1	2.458	40	24.97	40	25.84
B	19	46.84	1	2.464	50	31.27	30	19.42
C	19	46.95	1	2.470	60	37.61	20	12.98
D	14.25	38.16	0.75	2.045	45	31.14	40	28.65
E	14.25	38.69	0.75	2.036	55	37.89	30	21.39
F	14.25	38.77	0.75	2.041	65	44.89	20	14.29

**Table 3:** Mixing ratio using aluminum oxide and zinc oxide powder.

Type	Matrix (PDMS)				Filler					
	Base		Curing Agent		70 μm Al_2_O_3_		5 μm Al_2_O_3_		1 μm ZnO	
	wt%	vol%	wt%	vol%	wt%	vol%	wt%	vol%	wt%	vol%
B	19	46.84	1	2.464	50	31.28	30	19.42	0	0
B-ZnO 10	19	47.82	1	2.516	50	31.93	20	13.22	10	4.518
B-ZnO 20	19	48.84	1	2.570	50	32.61	10	6.749	20	9.228
B-ZnO 30	19	49.91	1	2.626	50	33.32	0	0	30	14.14
E	14.25	38.69	0.75	2.036	55	37.89	30	21.39	0	0
E-ZnO 10	14.25	39.59	0.75	2.084	55	38.77	20	14.59	10	4.988
E-ZnO 20	14.25	40.52	0.75	2.132	55	39.68	10	7.466	20	10.21
E-ZnO 30	14.25	41.50	0.75	2.184	55	40.64	0	0	30	15.68

**Table 4:** Mixing ratio using aluminum oxide and copper powder.

Type	Matrix (PDMS)				Filler					
	Base		Curing Agent		70 μm Al_2_O_3_		5 μm Al_2_O_3_		1 μm Cu	
	wt%	vol%	wt%	vol%	wt%	vol%	wt%	vol%	wt%	vol%
B	19	46.84	1	2.464	50	31.28	30	19.42	0	0
B-Cu 10	19	48.67	1	2.560	50	32.49	20	13.45	10	2.827
B-Cu 20	19	50.64	1	2.664	50	33.81	10	6.998	20	5.881
B-Cu 30	19	52.78	1	2.777	50	35.24	0	0	30	9.196
E	14.25	38.69	0.75	2.036	55	37.89	30	21.39	0	0
E-Cu 10	14.25	40.36	0.75	2.124	55	39.52	20	14.87	10	3.126
E-Cu 20	14.25	42.17	0.75	2.220	55	41.30	10	7.772	20	6.531
E-Cu 30	14.25	44.16	0.75	2.324	55	43.25	0	0	30	10.26

## Data Availability

Data is contained within the article.
